# Three‐Unit Prosthesis Supported by a Single Implant With Two Cantilever Extensions in the Posterior Region (T‐Bridge): Case Report With a 2‐Year Follow‐Up

**DOI:** 10.1002/ccr3.71777

**Published:** 2025-12-30

**Authors:** Jésio Rodrigues Silva, Cláudio Rodrigues Leles, Leuçon de Oliveira Moura‐Neto, Urs Brägger, Martin Schimmel, Manrique Fonseca

**Affiliations:** ^1^ School of Dentistry Federal University of Goias Goiania Goias Brazil; ^2^ Department of Reconstructive Dentistry and Gerodontology, School of Dental Medicine University of Bern Bern Switzerland; ^3^ Division of Gerodontology and Removable Prosthodontics University Clinics of Dental Medicine, University of Geneva Geneva Switzerland

**Keywords:** case report, dental implants, dental prosthesis design, fixed dental prosthesis, implant‐supported dental prosthesis

## Abstract

This report describes the rehabilitation of short‐span posterior edentulous spaces using a single morse‐taper implant supporting a porcelain‐fused‐to‐metal fixed prosthesis with bilateral cantilever extensions (T‐Bridge). The 2‐year clinical and radiographic follow‐up demonstrated stable peri‐implant tissues and satisfactory functional performance, without mechanical complications. Although the outcomes were favorable in this particular case, this approach should be considered with caution, and further clinical studies are necessary to confirm its predictability and long‐term applicability.

**Key Clinical Message**

A single implant supporting a three‐unit prosthesis with mesial and distal cantilevers may be a viable option in carefully selected posterior cases. Adequate bone quality, precise positioning, short cantilevers, and controlled occlusion are essential. Evidence remains limited, and findings should be interpreted cautiously until larger studies are available.

## 1 Background

Implant‐supported prostheses are a widely accepted treatment modality for the rehabilitation of edentulous spaces, particularly in posterior regions where functional and biomechanical demands are exceptionally high [1]. Conventional treatment protocols typically recommend placing one implant per missing tooth or designing short‐span fixed partial prosthesis supported by at least two implants [2]. However, these approaches are not always clinically feasible due to various anatomical limitations, including reduced bone height in the posterior maxilla or dangerous proximity to critical structures such as the mandibular canal. These situations often require additional complex procedures, such as bone grafting, which significantly increases treatment complexity and patient morbidity [3].

Moreover, the substantial financial cost associated with multiple implant placement may severely restrict treatment options for many patients, creating a clear need for alternative strategies that successfully balance biological soundness, functional excellence, and economic accessibility [1, 4, 5].

In this context, a treatment approach involving a three‐unit implant‐supported fixed dental prosthesis retained by a single implant strategically positioned within the edentulous span has been reported in the literature, including descriptions of unilateral or bilateral cantilever extensions [6, 7, 8]. Although this concept is not new, evidence remains scarce, particularly regarding posterior mandibular applications, where biomechanical demands are high and long‐term clinical data are still limited.

This case report presents the treatment of a short‐span edentulous area using the “T‐bridge” approach and the subsequent clinical and radiographic outcomes, aiming to contribute an additional clinical example to the limited literature available, while recognizing that further studies are required before broader clinical conclusions can be drawn.

## 2 Case Report

### 2.1 Case Presentation

A 43‐year‐old male patient in good systemic health (ASA I) presented at the research center for prosthetics and implants (NPPI) clinic, Federal University of Goias, seeking comprehensive oral rehabilitation. His primary concern included significant difficulty with mastication and eating caused by missing posterior teeth, mild discomfort in the edentulous region, and notable esthetic concerns affecting his confidence. The patient was a nonsmoker, and his comprehensive medical history revealed no contraindications for oral surgery.

**FIGURE 1:**
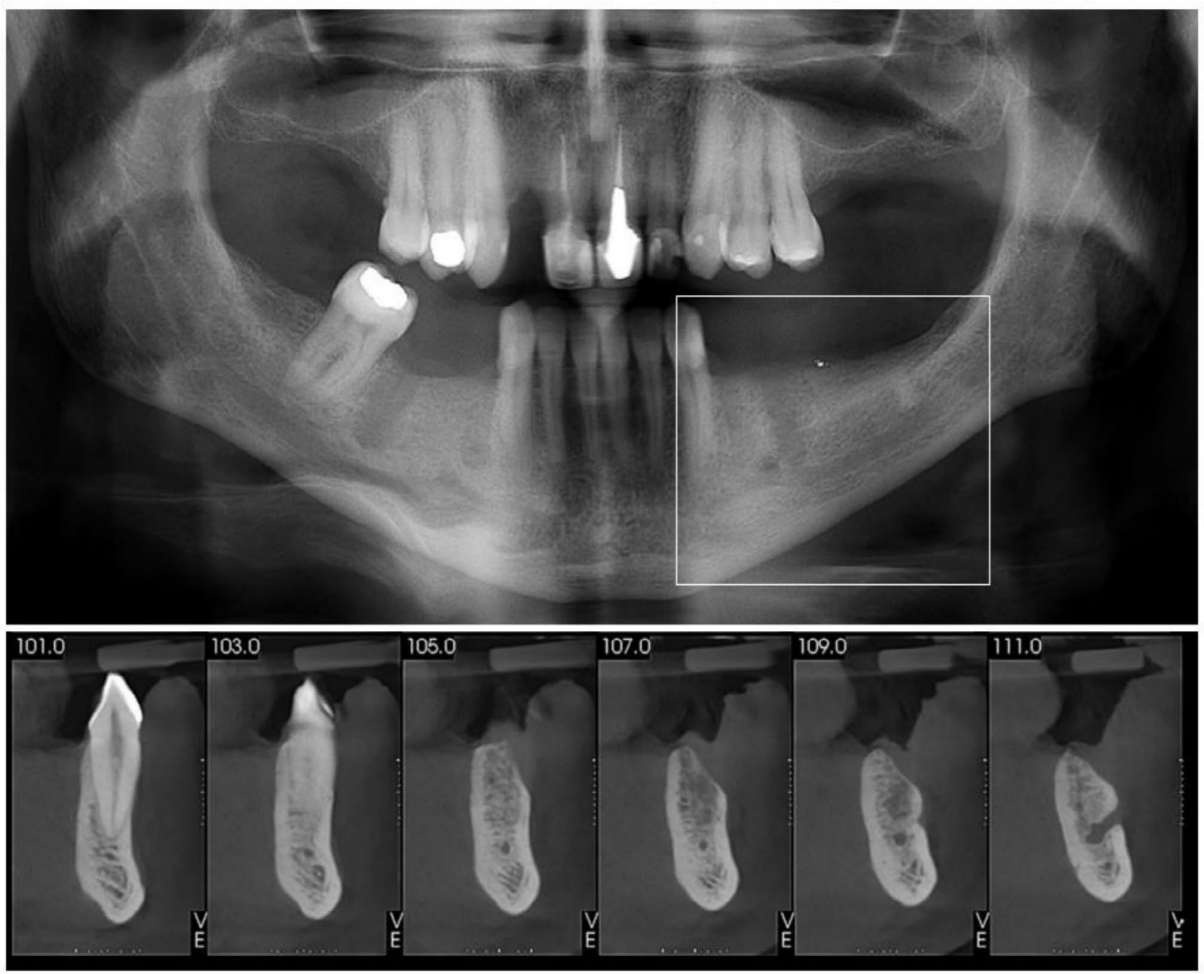
Initial radiographic and CBCT aspect.

Comprehensive clinical examination revealed a mandibular edentulous space corresponding to teeth #34, #35, and #36 on the left side, while the remaining teeth appeared to be in good overall condition. The opposing dentition was natural and presented in a healthy state, with adequate periodontal support. Radiographic evaluation, including intraoral periapical radiographs, panoramic radiography, and cone‐beam computed tomography, confirmed sufficient bone height and favorable anatomy for successful implant placement using the T‐Bridge concept (Figure 1).

At baseline, the patient presented mutually protected occlusion, no temporomandibular disorders, and no parafunctional habits such as bruxism or clenching. Previous dental records indicated that several teeth (including #34, #35, #44, and #45) had been extracted 1 year earlier as part of oral rehabilitation planning.

CBCT evaluation revealed adequate bone width and Type II bone density in the #35 region, and therefore a single implant was selected for this area. Further implant treatments were provided accordingly (teeth #12, #45, and #45), but were not detailed in this report. Additionally, the patient reported significant financial limitations and explicitly opted to avoid additional implants and surgical procedures whenever a less invasive alternative was acceptable.

The patient consented to the treatment provided after being fully informed about the treatment plan and the related procedures. Furthermore, a written informed consent was obtained from the patient for the publication of this case report.

### 2.2 Surgical Procedure

The implant surgery was performed under local anesthesia with 4% articaine and 1:100,000 epinephrine. Following antisepsis with 2% chlorhexidine externally and 0.12% chlorhexidine intraoral rinses, a full‐thickness mucoperiosteal flap was elevated. Sequential osteotomy was performed strictly following the manufacturer's recommended drilling protocol based on the assessed bone density and specific implant dimensions. A freehand surgery was performed with the aid of a surgical stent to assist in drilling procedures for correct implant positioning.

A single 4.3 × 8 mm morse‐taper implant (Max CM Cônico, Intraoss, Itaquaquecetuba‐SP, Brazil) was inserted in the region of tooth 35, which was strategically placed at the center of the edentulous space to support the planned three‐unit iFDP. The implant achieved an insertion torque of 30 Ncm, indicating adequate primary stability. A 3.5 mm healing abutment was positioned at the mucosal level for a single‐stage surgery.

The surgical flap was sutured with 5‐0 nylon sutures, and comprehensive postoperative care was provided, including ibuprofen 400 mg, amoxicillin 500 mg, dipyrone 500 mg, and 0.12% chlorhexidine mouthwash, along with detailed oral hygiene instructions for optimal plaque control.

**FIGURE 2:**
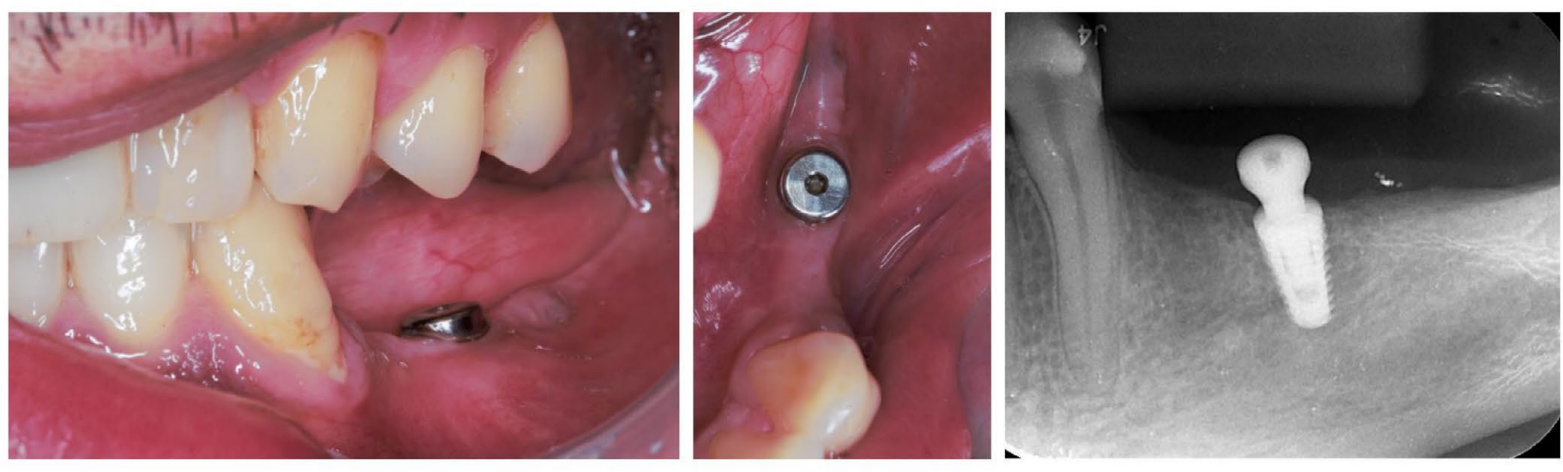
Clinical and radiographic aspects in the 2‐week recall visit.

A conservative healing period of 6 months was observed to allow osseointegration. Prior to the prosthetic phase, implant stability was confirmed by resonance frequency analysis, showing an ISQ value of 80, with no clinical signs of inflammation, infection, or mobility (Figure 2).

### 2.3 Prosthetic Rehabilitation

The prosthetic treatment plan consisted of fabricating a three‐unit porcelain‐fused‐to‐metal T‐Bridge supported by a single implant in position 35 and with bilateral cantilever extensions to positions 34 and 36. Prosthetic procedures included a conventional open‐tray impression at the implant fixture level, accurate bite registration, and alginate impression of the antagonist jaw for proper articulation. In the laboratory, master models were fabricated, and the metal framework was manufactured. A one‐piece abutment (Max CM Cilindro/Ucla, Intraoss, Itaquaquecetuba‐SP, Brazil) was used for the manufacturing of the metal framework of the definitive iFDP.

During the try‐in appointment, the metal framework fit was carefully evaluated, the interocclusal space required for ceramic was verified, and a periapical radiograph was taken to ensure passive fit of the new FDP. Subsequently, a meticulous shade selection was performed to achieve optimal esthetic integration with the existing dentition.

**FIGURE 3:**
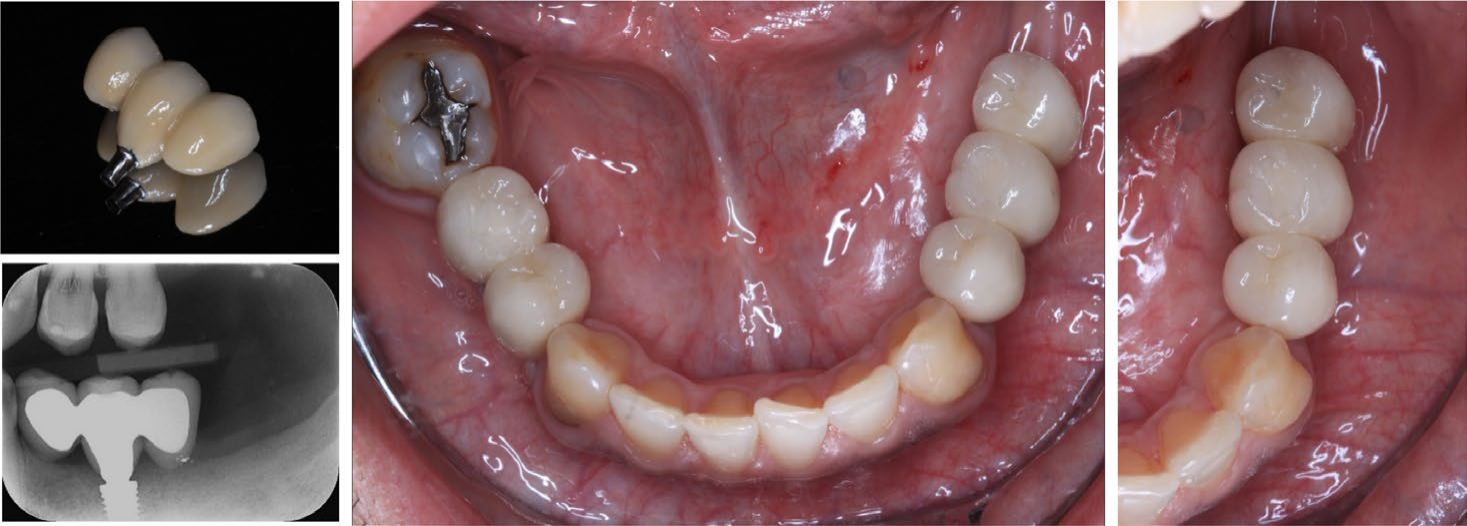
Clinical and radiographic aspects of the T‐Bridge prosthesis.

The final prosthesis was designed as a screw‐retained restoration featuring two extensions with premolar width dimensions, each approximately 7–8 mm in length. The prosthesis was delivered with a torque of 35 Ncm (Figure 3).

Special attention was given to occlusal adjustments to eliminate excursive contacts on both cantilever extensions. In maximum intercuspation, light contacts were retained primarily over the central fossa region to minimize loading effects on the extensions. The final occlusal relationship remained mutually protected, with canine guidance in lateral excursions.

The screw channel was sealed temporarily with Teflon (TDV, Pomerode, Santa Catarina, Brazil) and Telio (Ivoclar Vivadent, Barueri, São Paulo, Brazil).

A comprehensive follow‐up was scheduled 1 week postdelivery, during which the occlusal contacts and torque were re‐evaluated and controlled. Following this assessment, the screw access hole was definitively sealed using composite resin. No modifications to the original treatment plan were necessary throughout the prosthetic rehabilitation phase.

### 2.4 Follow‐Up and Outcomes

Since delivery, the patient followed a maintenance program including a professional cleaning twice a year and oral hygiene instructions. Throughout the follow‐up period, the patient demonstrated excellent compliance. Comprehensive clinical and radiographic evaluations conducted over a full 2‐year period consistently demonstrated stable peri‐implant tissues, complete absence of bone loss, and no prosthesis mobility observed.

During follow‐up appointments, a standardized clinical protocol was applied, including periodontal probing around the implant‐supported restoration, inspection for inflammation, and evaluation of bleeding on probing or suppuration. No bleeding, suppuration, or peri‐implant pathology was detected at any visit, and radiographic assessment revealed no evidence of marginal bone loss.

The patient reported high satisfaction with both the esthetic appearance and functional performance of the restoration, experienced no dietary restrictions, and maintained full compliance with all scheduled follow‐up visits.

The only minor observation during the follow‐up period was slight pigmentation of the composite resin covering the screw access, which is considered an expected occurrence in screw‐retained prostheses and does not affect the overall success of the treatment.

**FIGURE 4:**
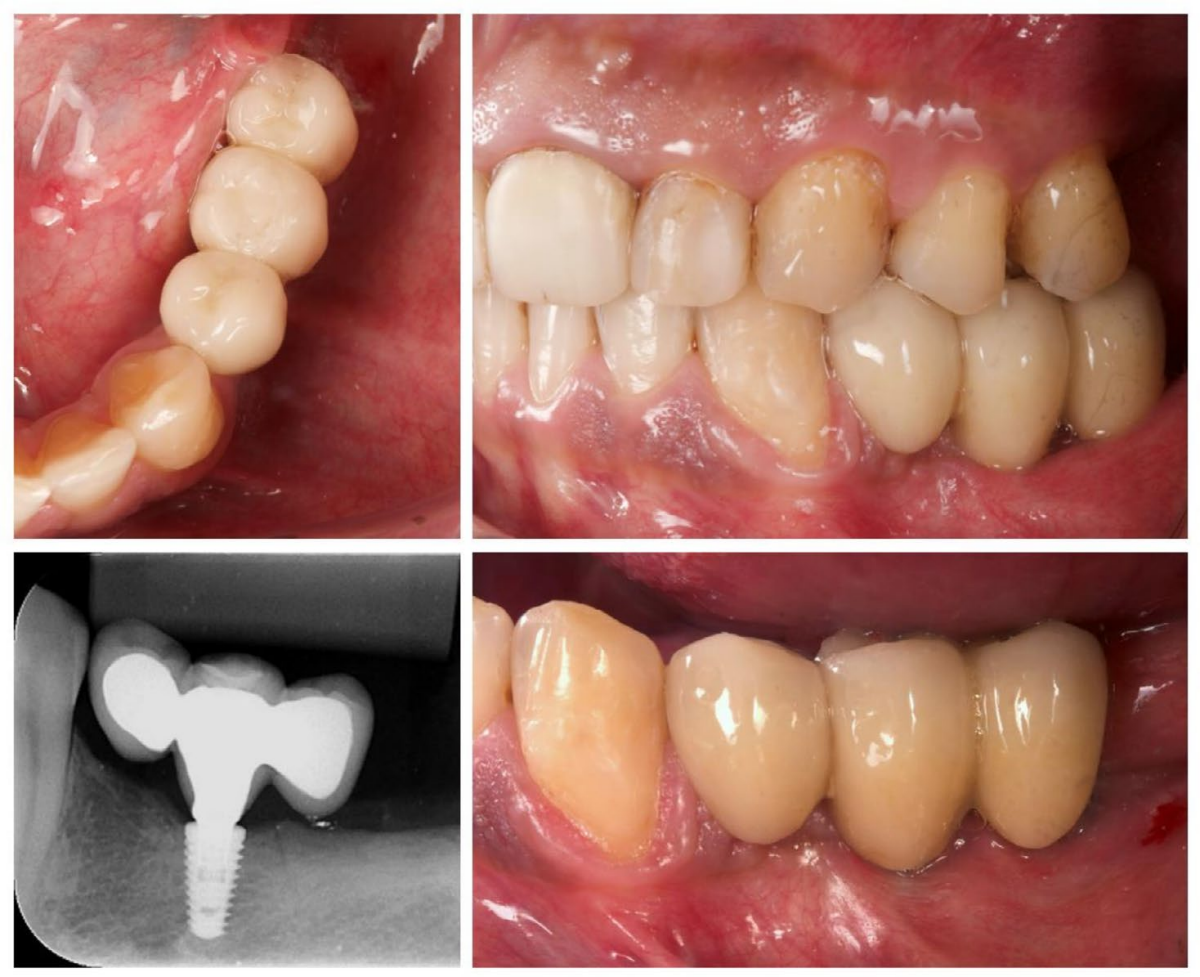
Clinical and radiographic aspects at the 2‐year follow‐up visit.

The 2‐year follow‐up visit showed satisfactory soft tissue health and occlusal stability. No prosthetic complications were observed during the follow‐up period, and oral hygiene was considered acceptable. The patient reported being fully satisfied with the treatment provided. Figure 4 reveals clinically sound peri‐implant tissues and no signs of peri‐implant bone changes.

## 3 Discussion

The case successfully demonstrates the clinical feasibility of an innovative treatment approach for rehabilitating edentulous areas in the posterior mandible, with comprehensive documentation over a 2‐year follow‐up period. However, rather than representing a novel technique, this approach has already been described in the literature [6, 7, 8], and the present report contributes only an additional clinical example, particularly in posterior mandibular sites where evidence remains limited [6, 7, 8]. The significant advantages of this treatment modality include reduced costs and patient discomfort related to implant surgery, ease of oral hygiene maintenance, and suitability for cases in which placement of multiple implants is anatomically or financially limited [4, 9, 10].

During the2‐year follow‐up period, the treatment demonstrated favorable clinical outcomes, radiographic peri‐implant findings, and positive patient‐reported results, suggesting both the feasibility and effectiveness of this approach in appropriately selected cases. Nevertheless, the conclusions of a single clinical case should be interpreted cautiously, and no generalized statements or clinical recommendations should be drawn from this isolated experience. Future longitudinal studies incorporating larger patient cohorts are necessary to provide robust evidence for the broader validity of this treatment concept, including quantitative assessment instruments for patient‐reported outcomes that could provide more precise information regarding patient satisfaction and the overall impact of this treatment modality on quality of life for partially edentulous patients requiring three‐unit iFDPs.

Mild biofilm accumulation was noted during routine follow‐up appointments, highlighting the importance of professional guidance and patient education in all implant‐supported rehabilitations. No clinical or radiographic signs of peri‐implant pathology were detected throughout the follow‐up period. The T‐Bridge design may actually offer advantages for hygiene access compared to three‐unit iFDPs supported by two implants, where interdental cleaning typically presents greater challenges [11].

Posterior mandibular regions may present unique challenges related to bone height in certain clinical situations. However, in the present case, CBCT evaluation revealed adequate bone width and Type II bone density at site #35, allowing placement of a single implant without grafting, which contrasts with the contralateral side that exhibited vertical deficiency and required augmentation. Additionally, the patient presented financial limitations and preferred to avoid additional surgical procedures, which influenced the treatment plan.

From a biomechanical perspective, replacing each missing tooth with an individual implant is theoretically ideal; however, this is not always feasible due to anatomical constraints, financial limitations, or patient preference [10, 12]. Single‐implant three‐unit prostheses with bilateral cantilevers impose biomechanical challenges, including increased lever‐arm forces and potential stress concentration [9, 10, 12, 13, 14]. In the present case, both cantilevers measured approximately 7–8 mm (premolar size), which is consistent with recommendations that shorter extensions may reduce bending moments [9, 10, 12, 13, 14].

When planning implant‐supported rehabilitations, particularly in biomechanically demanding posterior regions, clinicians must also carefully consider the potential influence of parafunctional habits such as bruxism or clenching [12]. Importantly, this patient exhibited no parafunctional habits, and mutually protected occlusion was established, eliminating eccentric contacts on the extensions and favoring axial loading, which may have contributed to the absence of mechanical complications.

Regarding occlusal considerations, implant‐supported FDPs in posterior regions require particularly careful attention to force distribution and direction. The presence of oblique or lateral forces is biomechanically detrimental and should be minimized whenever possible, whereas forces transmitted perpendicular to the implant body along the long axis tend to be significantly less harmful to the supporting bone [10, 13]. Within this biomechanical context, the occlusal design of a T‐Bridge should aim to position the supporting implant in the most central location possible, ensuring that functional forces are directed toward the center of the restoration, closer to the central fossa, and less concentrated on the potentially vulnerable distal or mesial extremities.

Recent in vitro and finite‐element studies have indicated that single‐implant‐supported three‐unit prostheses may exhibit lower fracture resistance than two‐implant‐supported FDPs and may be more susceptible to mechanical complications such as screw loosening or veneering fractures, especially when cantilevers extend mesially and distally [7, 8, 9, 10, 12, 13, 14]. Nevertheless, short cantilever length, proper implant positioning at the center of the edentulous span, and absence of parafunction were favorable conditions in this specific case.

The clinical success observed in this case can be attributed to adequate bone quality in the posterior mandible, appropriate implant positioning, and the rigid splinting design of the T‐Bridge, which likely enhanced load distribution [9, 10, 12, 13, 14]. In this specific case, the absence of mechanical complications over 2 years suggests that this approach may be biomechanically acceptable under favorable conditions. However, the mechanical behavior observed here should not be generalized or interpreted as evidence of predictability, and additional clinical studies with standardized follow‐up protocols are required.

## 4 Conclusion

The T‐Bridge concept observed in this clinical case suggests that, in carefully selected posterior situations, a single strategically positioned implant may support a three‐unit prosthesis under favorable biomechanical conditions. This approach represents a potential alternative in implant‐supported rehabilitation. However, the evidence from a single case is inherently limited and should be interpreted cautiously, and further clinical studies with larger samples and long‐term follow‐up are necessary to determine the predictability and broader applicability of this treatment modality.

## Author Contributions

**Jésio Rodrigues Silva:** investigation, methodology, project administration, resources, writing – original draft, writing – review and editing. **Cláudio Rodrigues Leles:** conceptualization, data curation, investigation, supervision, validation, writing – original draft, writing – review and editing. **Leuçon de Oliveira Moura‐Neto:** data curation, investigation, methodology, project administration, resources. **Urs Brägger:** conceptualization, validation, visualization, writing – original draft, writing – review and editing. **Martin Schimmel:** conceptualization, investigation, methodology, validation, writing – original draft, writing – review and editing. **Manrique Fonseca:** conceptualization, data curation, formal analysis, investigation, methodology, validation, writing – original draft, writing – review and editing.

## Funding

The authors have nothing to report.

## Data Availability

Data from this study may be provided under reasonable request.
